# Impact of the COVID-19 Pandemic on Infectious Diseases in Brazil: A Case Study on Dengue Infections

**DOI:** 10.3390/epidemiologia3010009

**Published:** 2022-03-02

**Authors:** Federico Borre, Juliette Ildiko Borri, Yuval Zoy Cohen, Mariana Gasparoto, Tsewang Bhumchok Gurung

**Affiliations:** Global Studies Institute, Université de Genève, 1211 Geneva, Switzerland; federico.borre@etu.unige.ch (F.B.); juliette.borri@etu.unige.ch (J.I.B.); tsewang.gurung@etu.unige.ch (T.B.G.)

**Keywords:** Brazil, COVID-19, dengue, emerging infectious disease, pandemic response

## Abstract

Brazil is known for being a breeding ground for emerging infectious diseases (EIDs), such as Zika, dengue, and chikungunya. Given that it has been one of the countries most affected by the SARS-CoV-2 pandemic, this article aims to analyze the impact that the COVID-19 pandemic has had on the burden of infectious diseases in Brazil, especially that of dengue. Brazil is a unique territory with a heterogeneous population living in a tropical, wet climate favorable to infectious diseases. In addition, despite being one of the largest emerging economies in the world, the country has been exposed to political instability and a public health system that suffers from large funding shortfalls and a lack of coherent regulation. The findings from this study are multilayered. Firstly, as cases of COVID-19 rose at the start of the pandemic, cases of dengue declined drastically. This may be due, in part, to factors such as seasonal climate and distancing measures. Furthermore, the findings indicate that the diversion of resources away from dengue and other infectious diseases, and mobilization for COVID-19 testing and treatment, likely resulted in a serious underreporting of dengue. While Brazil has incorporated some of the lessons learned from past EID experience in responding to the COVID-19 pandemic, the analysis highlights how the country’s structural problems present pitfalls in the epidemiological fight. It was concluded that in a country such as Brazil, where infectious disease outbreaks are only a matter of time, pandemic preparedness should be prioritized over pandemic response.

## 1. Introduction

The emergence of a new coronavirus, SARS-CoV-2, in 2019 led to a global pandemic of unprecedented scale and reach. In the last decade, emerging infectious diseases (EIDs) have been increasingly recognized as a global threat, as evidenced by the development of pandemic preparedness concepts such as the World Health Organization’s Disease X category (the “known unknown”) and work carried out by organizations such as the Coalition for Epidemic Preparedness Innovations (CEPI). To effectively prevent and control EIDs, it is critical to understand the nebulous factors and systems which lead to their appearance and spread [1]. No pathogen can be predicted before its appearance; however, the global community can take steps to note patterns in origin and spread and incorporate them into the design of disease surveillance programs [2].

Brazil has many key characteristics making it prone to such EID outbreaks. Populous and clustered cities, tropical regions, a rising rate of deforestation, high diversity in animal hosts, and expansion of anthropogenic activities are major drivers of infectious disease outbreaks, especially arboviruses [3]. While COVID-19 could not have been predicted exactly, Brazil’s uniquely high susceptibility to new disease outbreaks begs the question: How did Brazil’s experience preventing and controlling other EIDs shape its response to COVID-19? In the last five years alone, Brazil has experienced multiple infectious disease crises—an overwhelming dengue outbreak in 2019, off the back of the 2015–2017 Zika epidemic, which saw cases worldwide. Despite having previous experience with EIDs and pre-existing disease monitoring infrastructure and control programs, Brazil has still been incredibly impacted by the COVID-19 pandemic.

The aim of this paper is to describe the current situation in Brazil and how the COVID-19 pandemic has impacted the trajectory of other infectious diseases in the country, specifically dengue. Our research focused on dengue due to its consistency as a public health threat in Brazil and its sharp rise in cases immediately preceding the pandemic. This case study will first present background on the social, economic, political, and environmental factors that have impacted Brazil’s emerging infectious disease responses. We will then give an overview of the epidemiological situation and contextualize Brazil’s response to COVID-19 with its most recent experiences with epidemics of dengue virus (DENV) and Zika virus (ZIKV). Finally, this case study will discuss the effect COVID-19 has had on multiple facets of dengue prevention and control and reflect on lessons learned from past outbreaks on how to manage larger epidemics such as COVID-19.

## 2. Materials and Methods

This case study is a descriptive and analytical review of scientific literature, supplemented with gray literature and news sources due to the ever-evolving COVID-19 situation. The research team made use of Google Scholar, PubMed, Medline, Scielo, and ResearchGate, using the following keywords: “Brazil; census; population; politics; geography; demography; economy; socioeconomic distribution; vector ecology; COVID-19; dengue; Zika; emerging infectious disease; EIDs; pandemic response; pandemic preparedness; pandemic crises; *Sistema Único de Saúde*; *Programa de Combate à Dengue*; Bolsonaro; anti-science; anti-vaccination; COVID-19 cases; COVID-19 deaths; COVID-19 epidemiological curve; community strategies; social distancing; government intervention; nonpharmaceutical interventions; vaccines; testing capacity; underreporting; COVID-19 timeline”. A comprehensive review of five official Brazilian websites was completed to assess the most recent policies enacted, among them the Ministry of Health, IBGE (Brazilian Geography and Statistics Institute), the Brazilian Senate, LocalizaSUS, and the “Portal da Transparência”, as well as the Pan-American Health Organization and World Health Organization websites. We also used relevant news from the local and international press.

The searches were mainly in English and Portuguese. Scielo was searched to try and mitigate bias towards English and find sources from the Brazilian and Latin American research communities. Despite our efforts, not all researchers were able to review the Portuguese literature, which can still be considered a limitation of this study. The data were obtained from primary and secondary literature sources. Due to the descriptive nature of this study, no hypotheses were tested, and an analysis is provided in the Discussion section.

## 3. Case study

### 3.1. Case Presentation

#### 3.1.1. Demography

Brazil is the most populous country in the Latin American region, with just under 213 million inhabitants in 2020, close to double the population of the second most populous country, Mexico [4]. The Southeast is by far the most populous region with 89.63 million inhabitants, followed by the Northeast and South, while the Central-West and North regions are the least populated. About 40% of the urban population is concentrated in larger metropolitan areas such as São Paulo, Rio de Janeiro, Porto Alegre, Salvador, and Manaus [5,6].

During the 1970–80s, accelerated urbanization produced overcrowded cities with significant challenges, such as slums (*favelas*) and poor public health conditions [7]. The WHO/UNICEF estimates that close to 15 million Brazilians in urban areas do not have access to safe drinking water in their homes; additionally, 25 million people in rural areas have limited access to safe drinking water [8]. The most affected populations include Indigenous peoples and those living in *favelas* [8]. Such conditions make it difficult for individuals to follow infectious disease prevention recommendations. In the case of dengue, a strong recommendation is to remove stagnant water from around and inside the home, as this is a breeding ground for mosquitoes. Individuals living in areas where water is unavailable on-demand and thus needs to be stored find difficulty in heeding this recommendation. In the case of COVID-19, hand washing and social distancing are major challenges where water is not only scarce but also unclean. Additionally, urban mobility directly affects the population’s health regarding the transmission and spread of diseases. A significant part of the population lives in the peripheral zones of Brazil’s 50 biggest cities, making them more easily exposed during more extended periods on public transit [9].

These mobility difficulties also affect Indigenous populations’ access to medical facilities such as health posts, medications, and vaccines. They are an at-risk group, having had a history of being disproportionately burdened by infectious diseases [10]. During the H1N1 influenza pandemic in 2009, the death rate was 4.5 times higher among the Indigenous population than other ethnic groups and 6.5 times higher during the COVID-19 pandemic [11]. The same disparity exists among *Pardo* and Black Brazilians, who present a higher risk of death from COVID-19 infection than other racial groups.

The risk of death from COVID-19 is also higher in the northern part of Brazil than in the central and southern parts of the country, likely due to the fact that this part of Brazil has the highest proportion of vulnerable and at-risk populations [12]. Finally, Brazil has one of the highest inequalities in the world in regard to socioeconomic class: only 3% of the population is in the upper socioeconomic class. The North and Northeast regions present the highest levels of poverty and inequality—around 40% of the population is in the bottom socioeconomic classes. These numbers have worsened due to the pandemic crisis [13].

#### 3.1.2. Geography

Mainly situated in the southern hemisphere, Brazil is the largest country in South America and the fifth worldwide. With its territory divided into five large geographical regions called North (*Norte*), Northeast (*Nordeste*), Central-West (*Centro-Oeste*), Southeast (*Sudeste*), and South (*Sul*), Brazil has very heterogeneous geography [14]. Brazil’s climate varies among equatorial, tropical, and subtropical, with an identified drier region in the Northeast. The country’s heaviest monthly precipitations are seasonal, from January to March, receiving 40–70 inches (1000–1800 mm) of rain annually [15,16]. At the regional level, climate and temperature have been defining elements in outbreaks of disease, especially those spread by mosquitoes [17].

Urban municipalities are the minority in Brazil: only 21% of Brazilian municipalities are classified as urban. The percentage of the rural area varies from 35% to 42% in the North, Northeast, and South [18]. The Southeast and Central-West regions, respectively home to the megacities of Rio and São Paulo and the Federal District, set themselves apart with the lowest number of rural municipalities. Even though rural and intermediate areas are the majority, most of the population is concentrated in urban areas. A clear example is the São Paulo macrometropolis, with more than 30 million inhabitants, or about 75% of the entire São Paulo state population. In total, 87% of the Brazilian population lives in urban areas [19].

Vector ecology is greatly influenced by climate and geography: in regions with higher rainfall and tropical climate, we see higher levels of mosquitos and, consequently, vector-borne diseases [17]. The South and Southeast regions, as well the Amazonian region in the North, have an optimal setting for mosquitoes to breed and circulate and are linked to greater outbreaks of arboviruses. In contrast, the Northeastern region, where there is less rainfall and the climate is milder, tends to report fewer infectious disease outbreaks. Brazil’s environment is host to several endemic EIDs, including dengue, Zika, yellow fever, and other arboviruses, as well as kinetoplastids, such as leishmaniasis and Chagas. Vectors such as *Aedes aegypti* mosquitoes are found all over the country but especially near megacities such as São Paulo. Mosquitoes are attracted to stagnant water, which is common in surrounding areas of cities and tropical rain forests.

#### 3.1.3. Economy

Brazil is one of the largest emerging economies in the world—the largest in South America and the 12th globally. Political instability and EID outbreaks continue to impact economic growth, particularly in the services and industry sectors, which make up 90% of the Brazilian GDP [20,21,22]. In recent years, the agriculture sector’s growth has been due to expanded soy plantations and cattle ranches, which have displaced populations to urban areas, favored deforestation, and caused a concentration of wealth, generating more social inequality. This lends itself to the proliferation of infectious diseases, especially in areas near the Amazon Forest and the Central-West, where we observe a concentration of big properties [23].

Between 2010 and 2020, the country witnessed the worst decade of recession in the last 100 years. In 2020, the COVID-19 pandemic imposed new levels of difficulty for Brazil’s already faltering economy, and the measures to contain the virus’ advancement particularly affected the service sector and informal workers [22]. The crisis raised the level of Brazilians in extreme poverty from 11% in 2019 to 16% in 2020. In an attempt to mitigate the financial repercussions of the pandemic, the Bolsonaro government launched the *Auxílio Emergencial* in April 2020, a program designed to help informal and unemployed workers who were not previously receiving help from the *Bolsa Família* assistance program, reaching 68 million people [24,25].

The significant challenges posed to the Brazilian economy by the country’s healthcare are not new. Diseases such as dengue, Zika, and chikungunya have historically burdened both the Brazilian health system and the national economy. In 2016, with an emerging epidemic of Zika concurrent with the country hosting the Olympic Games in Rio de Janeiro, Brazil spent BRL 2.3 billion on the management of the three arboviruses. Expenses ranged from the purchase of insecticides to medical treatment, in addition to the loss in income due to illness [26]. The average person loses 5 to 7 days of work after being infected with dengue fever, impacting companies’ production and interrupting independent or informal workers’ income [27], supporting a business case for the return of investment in public health measures to prevent outbreaks. A specific law establishing the minimum amount of investment in Public Health Actions and Services (ASPS) was enacted in 2006 due to the history of neglect by the politicians responsible for the decisions concerning the health system financing, resulting in underfunding [28,29]. The efficacy of this policy is yet to be determined, given the current struggle of the healthcare system.

#### 3.1.4. Politics

The current president, Jair Bolsonaro, a member of the Social Liberal Party, is in charge of Brazil’s pandemic response. His political career has been characterized by very strong and controversial stances, such as threatening the LGBTQ+ community’s rights, denying climate change, and appointing personalities who question scientific veracity [30]. Since the start of the pandemic, Bolsonaro has been criticized globally for downplaying the severity of the situation in Brazil, managing an extremely slow roll of both testing and vaccination, and prioritizing the economy over enforcement of lockdowns. He has made controversial statements, such as defining COVID-19 as a mere “*gripezinha*” and stating that 90% of people infected would not feel any symptoms [31]. While state and local authorities in Brazil were combating COVID-19 with solid measures, Bolsonaro denied the severity of the disease and undermined efforts to reduce transmission, often spreading misinformation and damaging trust in scientific evidence [32].

The *República Federativa do Brasil* is a federal presidential democratic republic divided into 26 federal states and 1 federal district [33]. Due to this decentralization and lack of support from the executive office, there exists a sense of incoherency between the federal and state governments in Brazil, as well as ambiguity over budget allocations and responsibilities, which has hampered quick and decisive action. An enormous responsibility was placed on state governments to determine their own responses to the pandemic rather than having a unified national approach. This is not a new phenomenon; during the 2015-2017 Zika outbreak, political instability and distrust in the government hindered the epidemic response, along with historical political decisions to underfund the health system, leaving it unable to adequately manage and care for Zika and microcephaly cases [34]. Despite this example in the country’s recent history, the importance of coherent national strategies and an adequately funded healthcare system has been largely unrecognized. Nearly two years into the COVID-19 pandemic, it is clear that these two political issues are again an obstacle to controlling outbreaks.

#### 3.1.5. Brazil’s Healthcare System

Today’s public healthcare system in Brazil, the Unified Health System or *Sistema Único de Saúde* (SUS), is decentralized, which means that the federal government, states, and municipalities split the system’s decision making, participation, and funding [35,36]. Considered one of the largest public healthcare systems globally, SUS is responsible for all levels of care: from primary to emergency care, vaccination programs, sanitation, and epidemiological surveillance. However, the Brazilian Health System comprises public and private sectors and is divided into a distinct but interconnected network of three subsectors: the public subsector (SUS); the private subsector (for-profit and nonprofit); and the insurance and health plan sector. Only the public subsector offers prevention and curative care along with free medication. The other subsectors rely on the purchasing power of each citizen, highlighting stark social inequality, as only a quarter of the population is covered by private health insurance [35].

Although the creation of the SUS is considered a great success, its funding has been a source of debate and concern since its implementation. Despite the constitution’s clear outline of the SUS’s responsibilities and funding sources, the minimum amount of investment was established only one decade later and is arguably too low for it to complete its functions at an optimal standard [37]. Consequently, the SUS has had a history of underfunding and political neglect that has been brought to the forefront during the COVID-19 pandemic. Despite the financial challenges, the SUS implemented programs to combat serious public health issues, such as the HIV and the Dengue Fever Program (*Programa Nacional de Controle da Dengue*, (PNCD)) [37,38]. During the COVID-19 pandemic, the vaccination program has gained immense prominence. In spite of significant bottlenecks, vaccines have been distributed free of charge. By November 2021, 77.2% of the population had received at least one dose of the vaccine, 62.9% had been fully vaccinated, and 6.6% had received a third (booster) dose [39].

Since the beginning of the pandemic, all three levels of the government have struggled to compensate for the lack of resources, insufficient testing capacity, and other deficiencies of the SUS. Despite an investment of BRL 524 billion in 2020 alone, the health system still struggled to avoid multiple crises, such as the shortness of oxygen supply in the North region at the pandemic’s peak [40,41].

Moreover, it is essential to note that other infectious diseases have historically burdened the Brazilian healthcare system. Immediately before the COVID-19 outbreak in Brazil, the country was suffering from one of the highest spikes in dengue in the last 25 years. During this period, public hospitals and health units were operating over their capacity in many states. The system was overwhelmed—and authorities were heavily criticized for how they dealt with the crisis and their reluctance to learn from previous outbreaks. COVID-19 only exacerbated the underfunded SUS [42]. Control of dengue, Zika, and chikungunya demands a substantial investment annually; the SUS is responsible for vector control, notification of suspected cases, training of agents, awareness campaigns at the national level, and treatment. In 2016 alone, the economic impact caused by the arboviruses stood at about BRL 2.3 billion [26]. Moreover, the diverting of funds away from dengue and towards COVID-19 will likely have lasting impacts on the capacity of the SUS to carry out future dengue control programs.

### 3.2. Management and Outcomes of Emerging Infectious Disease Outbreaks in Brazil

#### 3.2.1. COVID-19: 2020–21

As the world nears the two-year milestone of the emergence of SARS-CoV-2, Brazil stands out as one of the most severely impacted countries in terms of morbidity and mortality. The epidemiological situation has quickly deteriorated since the disease was first laboratory-confirmed in Brazil on 26 February 2020 in São Paulo. With a cumulative death toll of 607,824 deaths as of 30 November 2021, Brazil is second to only the United States of America in this metric. When looking at the case-to-fatality ratio (CFR), Brazil has even beat the USA, which sits at 1.6% compared to Brazil’s 2.8% (this measure begs cautious interpretation as the true number of total cases is unknown) [43]. Brazil also became the epicentre of COVID-19 in Latin America with a reported total of 21,810,855 cases as of 30 November 2021, nearly six times the number of cases recorded in Argentina and Colombia, which have the next highest case numbers [43]. It is important to note, however, that at the height of the third wave in Brazil (24 June 2021), the country’s rate was 85,252.53 per million population, lower than both Argentina and Uruguay [43].

Following this first report, cases of COVID-19 spread through Brazil at an unprecedentedly fast pace due to a number of state-distinct reasons, including population density, socioeconomic conditions, and differences in how states enforced regulations, resulting in multiple, simultaneous epidemics throughout Brazil [44]. By July 2020, Brazil experienced its first peak, reporting around 45,000 cases and 1000 deaths per day. Transmission and deaths remained high following the spike, and cases did not begin trending downwards until September 2020—hitting an all-time low in early November. Off the back of this came an almost linear rise in daily cases to new heights in 2021. Brazil’s second wave peaked in March 2021, at levels nearly 50% higher than the first peak.

**Figure 1:**
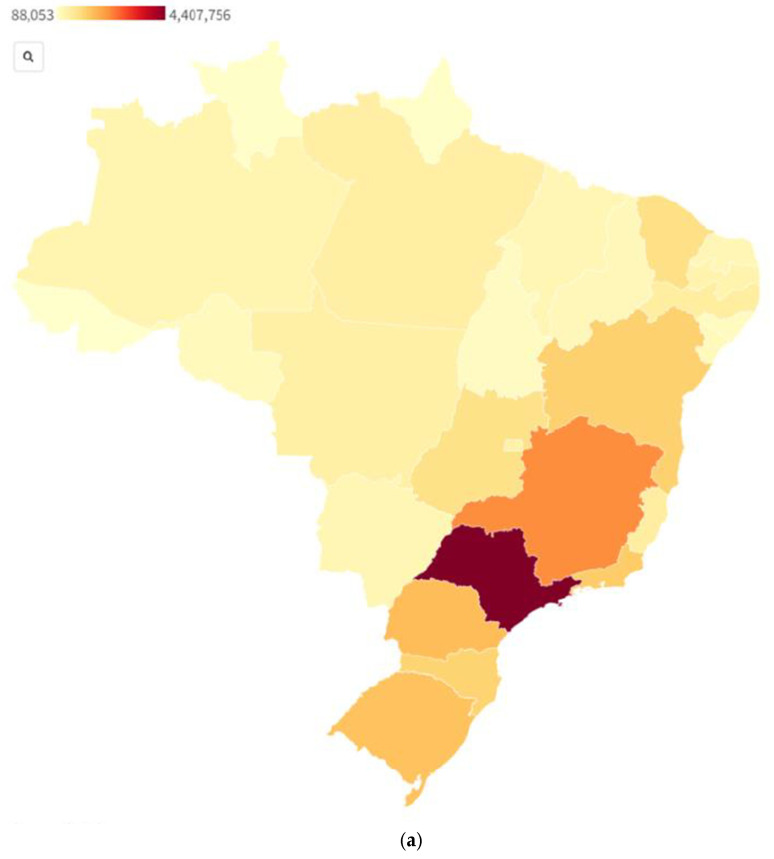

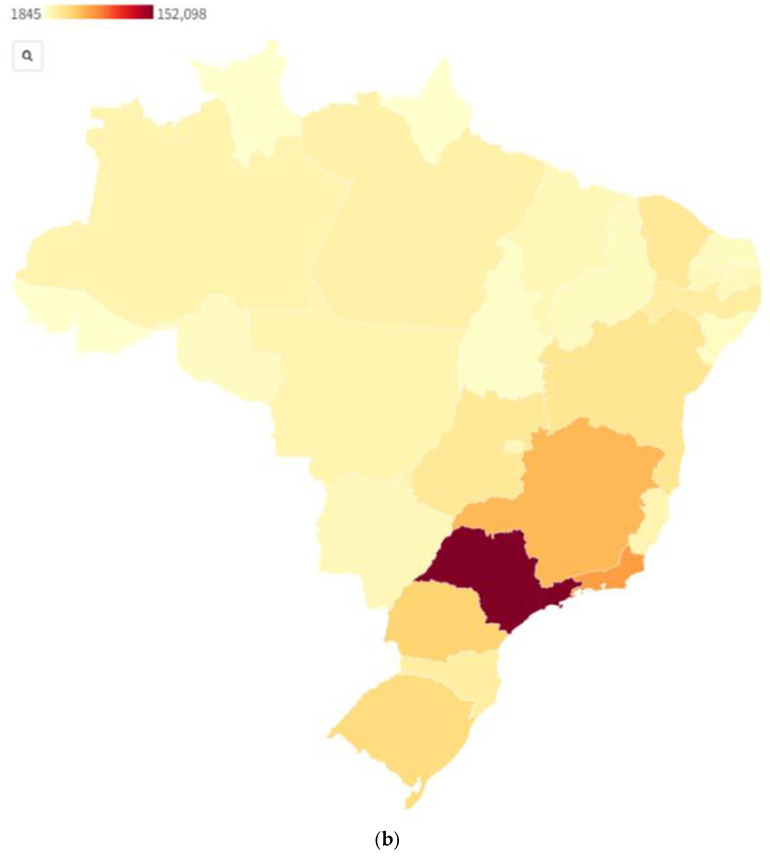
Confirmed COVID-19 cases (**a**) [48] and deaths (**b**) [49] by state as of 4 November 2021, Brazil.

Unlike in 2020, the decline observed directly after the peak did not last, with a third peak occurring in late June 2021, which saw equally high levels of cases. Influencing the epidemiological curve was the introduction of a new Variant of Concern (VOC), the Delta strain, with increased transmissibility of up to 50–100% [45]. On 27 March 2021, Brazil recorded its first case of the Delta VOC, which, by September of that year, would make up 90% of reported cases [45,46,47]. With vaccination campaigns ramping up, the epidemiological curve began to taper off as the fully vaccinated population grew four-fold from approximately 12.6% on 1 July 2021 to 56.4% by 31 October 2021 [43]. As of the last week of November 2021, daily cases remained above 9000 [43]. While COVID-19 has had a devastating impact at a national level for Brazil, the spread and burden of the disease have been unequally distributed across regions, states, and cities. Using publicly available data, this research team was able to map confirmed COVID-19 cases and deaths reported by Brazilian states as of 4 November 2021 in Figure 1 and Table 1.

As seen in Table 1 and Figure 1, São Paulo is the state with the highest numbers of reported COVID-19 cases and deaths, almost twice those of its neighboring states (Minas Gerais and Rio de Janeiro for cases and deaths, respectively). At a regional level, the Southeast has experienced over a third (39%) of all cases and close to half of all deaths (48%). In comparison, the North has recorded just 8.5% of cases and 7.7% of deaths. These numbers should be interpreted in a demographic context, with the Southeast having a larger population and containing cities such as São Paulo and Rio de Janeiro.

In the absence of safe and approved pharmaceutical products, the strategy to curb transmission of COVID-19 focused on nonpharmaceutical interventions (NPI) until safe vaccines and therapeutics were not only developed but also became more widely accessible. The aim of NPIs is to slow down the spread and transmission of COVID-19. In advance of new treatments being developed and registered for COVID-19, Brazil endorsed a regimen of unevidenced drugs including hydroxychloroquine and erythromycin under the name “kit-COVID” [31]. In Brazil, the implementation of NPIs has been uneven, uncoordinated, and generally unsupported at a federal level, which may have contributed to the rapid spread of the epidemic across the country [50]. COVID-19 was declared a public health emergency by Brazil on 3 February 2020 [51]; however, as previously mentioned, the federal response to COVID-19 lacked a nationwide social distancing policy, leaving state governments to play a decisive role in designing and enforcing NPIs. The federal response has primarily been shaped by the political leadership’s ideology, which has prioritized opening the economy and downplaying the severity of the pandemic and disease itself [52]. This manifested itself as well in the success, or lack thereof, of testing campaigns, leading to rampant underreporting. Brazil’s national testing strategy was fragmented between public and private efforts to scale-up diagnostic capacity [53]. Médecins Sans Frontières reported in June 2020 that testing was being rolled out at “an incredibly slow pace,” with Brazil reporting a fraction of the tests (7500 tests per million) of the US (74,927 per million) and Portugal (95,680 per million) [54].

By the end of March 2020, all states had adopted physical social distancing policies with varying levels of enforcement and effectiveness [55]. By April, some states had started relaxing their social distancing measures, a trend that continued until October 2020. Even as cases rose in late 2020 and early 2021, state governments did not impose stricter measures until March 2021, at the height of the second wave. Despite the situation being significantly worse than in the first wave of June 2020, social distancing policies were less strict than those previously adopted earlier in the pandemic [55]. While state governments adopted social distancing measures as a preventive strategy in March 2020, their strategy in 2021 tended to be more reactive, undermining the effectiveness of NPIs.

Another challenge was the environmental and socioeconomic context in which NPIs were being implemented. The types of measures used to combat COVID-19 are not always feasible or appropriate for urban settings in Brazil. With approximately 13 million Brazilians living in *favelas*, with cramped conditions and little access to clean water, it is difficult to adopt physical distancing measures, hygiene recommendations, and complete isolation or quarantine orders [56]. The extent to which NPIs are effective not only depends on public willingness and adherence but also the reality of living conditions that can potentially render social distancing infeasible.

While the COVID-19 pandemic has been an exceptional and unprecedented situation, Brazil has a history of dealing with emerging infectious disease outbreaks. Most recently, Brazil experienced outbreaks of dengue and Zika, for which they have developed response policies and strategies. A key difference to note between past outbreaks and the ongoing COVID-19 pandemic, however, is that Zika and dengue are both arboviruses, spread through a mosquito vector, while COVID-19 is a coronavirus that is airborne. This fundamental difference shapes policy to prepare for and respond to these types of outbreaks. Examining these outbreaks and, more importantly, Brazil’s response to them, is vital to better understanding the country’s response to COVID-19 and how the coronavirus impacted existing EID prevention and control programs.

#### 3.2.2. Dengue–2019

Dengue, an arbovirus, is a mosquito-borne viral disease that has rapidly spread throughout the world. There has been an eight-fold increase in cases in the last 20 years alone [57]. Dengue is largely found in tropical and subtropical climates—more specifically in urban and semi-urban areas—common in Brazil. Dengue virus (DENV) infections are mostly mild, with flu-like symptoms, and while mortality is low, infections can progress to severe disease and result in death. The virus places an extensive burden on healthcare systems that need to diagnose, manage, and treat DENV cases.

**Figure 2:**
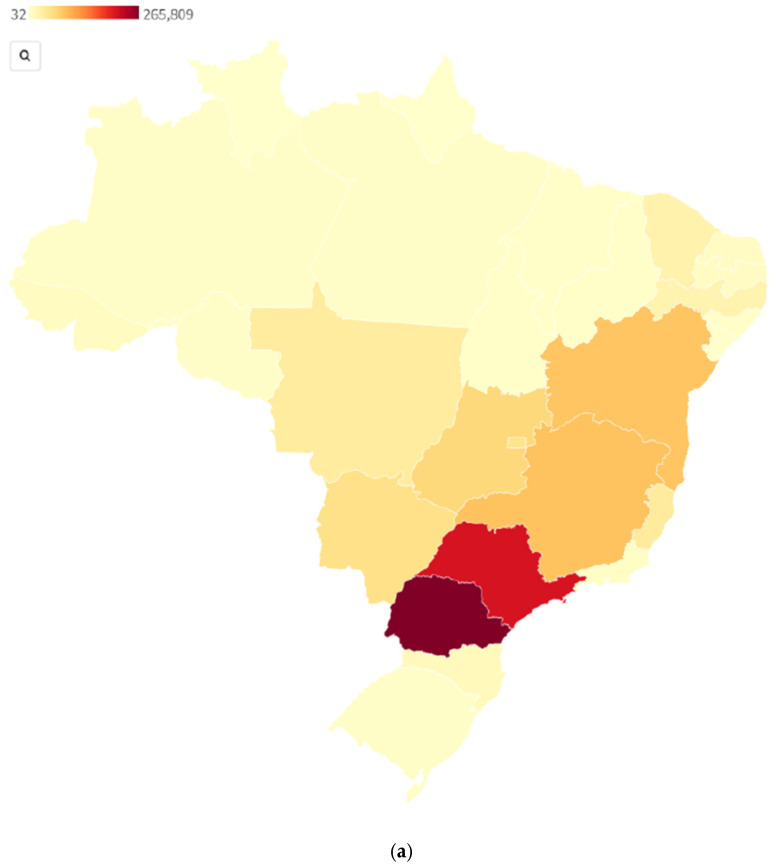

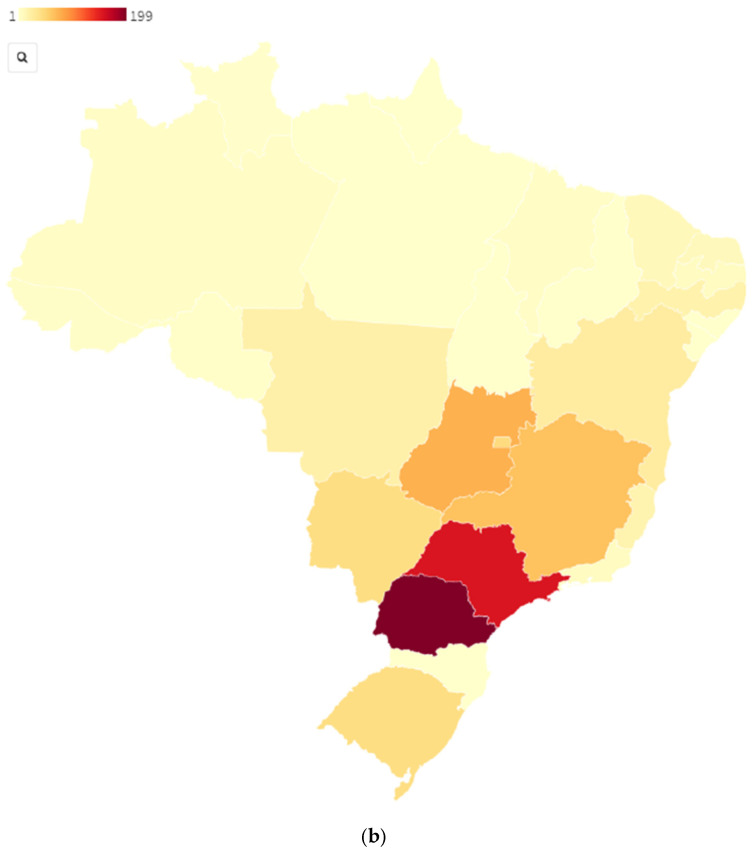
Probable dengue cases (**a**) [59] and confirmed deaths (**b**) [60] by state 2020, Brazil. Sources.

The most recent dengue outbreak in Brazil was in 2019, following a period of low transmission post-Zika, which is still yet to be fully understood. Brazil recorded over two million probable cases of dengue, including over 700 deaths, between January and November 2019, resulting in a cumulative national incidence rate of 711 cases per 100,000 population for 2019 compared to 107 per 100,000 population for the same period in 2018, representing a 573% increase [58]. The highest incidence rate was reported in the Central-West region (1248 cases per 100,000 population) and the lowest in the South (140 cases per 100,000 population) [58]. Just under two-thirds of notified cases in Brazil came from two federal units: Minas Gerais (2281 cases per 100,000 population) and São Paulo (1535 cases per 100,000 population). Figure 2 displays complementary information to Figure 1 for probable dengue cases and confirmed cases in terms of spread and mortality. While it provides an interesting perspective against the COVID-19 figures, it is not directly comparable given the differing period; the figures for dengue are given for 2020 only, while for COVID-19, they are cumulative as of 4 November 2021, which is a limitation.

Brazil has established a number of programmatic elements to manage emerging dengue cases. Dengue is a disease of compulsory notification in Brazil, which must be carried out through the Notifiable Disease Database (*Sistema de Informação de Agravos Notificáveis*, SINAN), meaning each confirmed case is reported to municipal health units [61]. The Federal Ministry of Health also has an established Brazilian Dengue Control Program (*Programa Nacional de Controle da Dengue*, PNCD). Additionally, Brazil has enacted legislation to integrate a legal framework into its vector management plan, allowing field activities to monitor and eliminate breeding sites [62]. Insecticide resistance is closely monitored, with the Ministry of Health purchasing insecticides and distributing them to municipalities. At a community level, prevention and control programs have been implemented with mixed efficacy. A review of multiple case studies in Brazil has found that while knowledge of dengue among communities is satisfactory, this does not necessarily translate into effective actions or entomological impact [63].

The Pan-American Health Organization (PAHO) provided advice to member states to prevent and manage future outbreaks, including strengthening disease surveillance systems and laboratory capacity for diagnosis. In retrospect, and with the experience of COVID-19, it is clear that testing capacity was not bolstered enough in response to deal with future emerging threats. In contrast, the SINAN notification system has been instrumental in data collection and formed the basis for COVID-19 data collection. While dengue outbreaks have not been experienced on the same level as COVID-19, there are similar critiques to be made about the effectiveness of community strategies and resourcing, which in both cases have put a strain on the public health system.

#### 3.2.3. Zika–2015–2017

Like dengue, Zika virus (ZIKV) is also a mosquito-borne arbovirus which is transmitted by the *Aedes aegypti* mosquito. The majority of cases remain asymptomatic, while a minority have a flu-like presentation. In October 2015, however, Brazil suspected a concerning association between ZIKV and microcephaly in newborns [64].

ZIKV cases in Brazil were first laboratory-confirmed in early 2015 in the Northeast. It was not until late 2015 that an uptick in babies born with microcephaly and other neurological disorders was linked to ZIKV [34]. Brazil declared a national public health emergency in November 2015, and in February 2016, the WHO declared these brain abnormalities a Public Health Emergency of International Concern (PHEIC) while emphasizing the possible connection between Zika and Guillain–Barré syndrome [34,64,65]. Despite the public health crisis, there was still much political and economic pressure to host the Olympic and Paralympic Games in Rio de Janeiro starting in August 2016. It appears now that the WHO’s declaration of a PHEIC was a way in which to act quickly and control the Zika outbreak and put pressure on Brazil to react with a strong policy response ahead of the country hosting the Games. The prioritization of the financial and diplomatic benefits of the Olympics in Rio is similar to what has been seen in 2021 with the most recent Olympic Games in Tokyo.

Brazil’s strategy during the Zika outbreak was based on three pillars: vector control, access to care for affected individuals, and research and development. This included the deployment of armed forces in *Aedes*-infested areas to eliminate potential mosquito breeding sites and distribute mosquito-repellent cream to pregnant women (which was later criticized for potential risks of active ingredients) [34]. Meanwhile, the epidemic was concentrated in one of the poorest areas of Brazil in the Northeast region, where there is a significantly low proportion of doctors per 1000 people. Furthermore, failed attempts at adequately funding the SUS left the healthcare system in a suboptimal position to deal with growing outbreaks [34].

Once again, parallels can be made between Brazil’s previous handling of emerging infectious disease epidemics and its COVID-19 response. Zika was on a slightly more comparable scale with COVID-19 than dengue due to its international spread as well as politicization.

### 3.3. Current Impact and Long-Term Influence of the COVID-19 Pandemic on Global and Public Health, Public Policy, and Healthcare System

As demonstrated above, the COVID-19 pandemic exacerbated the pre-existing weaknesses in Brazil’s financial, political, and public health systems. Since access to health insurance or private services depends on individual purchasing power, the prevailing social inequity manifested, for instance, in access to ICU beds. Until March 2020, SUS had an average of 1.4 beds for every 10,000 inhabitants, compared to 4.9 in the private network [41]. The same gaps in the public healthcare system existed within the individual regions; while the Southeast region had 2.7 ICU beds/10,000 inhabitants, the North had 0.9 beds/10,000 inhabitants, severely affecting access to intensive care for the Brazilians living there [41]. Moreover, in the first six months of the pandemic, the unmet need for ICU beds was higher in the North, Southeast, and Northeast than in the other regions of the country. Similarly, the in-hospital mortality rates and frequency of more invasive ventilation treatments were much higher in the Northeast region than in the South, even for patients under 60 years of age, highlighting a higher caseload of severe disease [66].

The disparity in ICU resources during the pandemic was only one symptom of the overwhelmed health system in Brazil. Despite the fact that at the beginning of the pandemic, certain regions were experiencing the effects of the pandemic more than others, by 2021, the shock to the health system was felt across all regions [67]. By March 2021, when COVID-19 cases peaked for a second time in Brazil, all but one of its 27 states were under stress, and ICU beds were being occupied at well over 90% capacity in the majority of states [67,68]. In addition to shortages in critical resources, such as ICU beds, medicine, and oxygen [69], the pandemic has had a significant impact on the wellbeing of healthcare workers, the full effects of which will only be fully discovered in time. Studies have reported increases in anxiety and depression among healthcare workers [70]. While cases have declined since the peak in March–June 2021, the effects of the pandemic on the mental health of healthcare workers in Brazil, as well as on the health systems in the individual states themselves, will likely be felt for many years to come—particularly as Brazil copes with the management of Zika, dengue, and other infectious diseases, the resources for which have been largely diverted to respond to the COVID-19 pandemic.

The pandemic has also exacerbated the economic situation of many people living in Brazil. While over one-fifth of the population was impoverished before COVID-19, the pandemic has pushed some of the middle and lower socioeconomic classes into the poorest class [71,72]. This has only worsened the existing disparities; as some classes have begun to recover, the poor are only getting poorer, and most of them consist of racial minorities [71,72]. As mentioned above, the areas in which the poorest populations live—crowded, urban areas with inconsistent access to clean water—are precisely the ones in which the spread of viruses is highest, resulting in a higher incidence of disease. Given that they were already disadvantaged and more likely to be unemployed or experiencing homelessness, these populations of Brazilian society were impacted the most by the pandemic, both in their socioeconomic status and their health [73].

The COVID-19 pandemic has also exacerbated many social and racial inequities that existed in Brazil prior to the pandemic. In August of 2020, researchers found that mortality rates from the virus were 250% higher for Indigenous people in the Amazon than for the general population, and incidence and mortality figures were being underreported by the government [74,75]. While disproportionate health outcomes are not unique to the most recent pandemic, COVID-19 has significantly intensified existing gaps in healthcare access among marginalized communities in Brazil by region, race, and socioeconomic status, as demonstrated above as well.

Finally, the COVID-19 pandemic has had and will likely continue to have a notable impact on the future of public health in Brazil. On the one hand, it has brought forward innovation and creative solutions. A prime example is telemedicine. Digital health was not necessarily new in Brazil before the outbreak of COVID-19, but it had not yet become mainstream. The Brazilian National Telehealth Network Program and the Telemedicine University Network were established in 2006 by the federal government in a move towards the innovation of the SUS and to increase interconnectivity and reach remote populations to improve quality of care [76]. By and large, however, these networks were not widely known to the general Brazilian public until the pandemic. Investment in telemedicine allowed cases of COVID-19 to be managed and treated virtually, showing quite a bit of promise for the future of healthcare delivery in Brazil [77]. The expansion of telemedicine could also positively impact surveillance and treatment of EIDs, particularly in remote areas.

In tandem, the emphasis on telemedicine and the restrictions on in-person care are suspected to have had a negative impact on the management of other diseases, including chronic, noncommunicable, and infectious diseases. Studies found that by August 2020, cervical and breast cancer screening decreased substantially compared to pre-pandemic rates, hospitalizations related to cancer treatment and surgery declined, and many patients with diabetes postponed their regular examinations [78,79,80]. These delays in care for illnesses where early detection and management are so crucial are expected to have long-term consequences on the burden of chronic disease in Brazil. Moreover, as previously noted, Brazil experienced the outbreak of COVID-19 while simultaneously managing dengue, Zika, and chikungunya, for which the government has set up surveillance and testing programs [81]. There is strong evidence to suggest that the diversion of resources towards COVID-19 had a substantial impact on the testing capacity dedicated to these infections, particularly given that many of them present symptoms similar to the novel coronavirus. Thus, underreporting of other infectious diseases in Brazil is highly likely, and the poor management of these diseases will have a lasting impact on their epidemiological curve in the coming years. This fact, coupled with the mismanagement of noncommunicable diseases during the pandemic, has the potential to exacerbate the already developing double burden of disease in Brazil [82].

## 4. Discussion

This paper has provided a multifaceted description of Brazil and its previous experience with emerging infectious disease outbreaks. We have discussed not only how COVID-19 has impacted each facet of Brazilian society but also how it ties back to previous EID outbreaks and lessons learned. Here, we will discuss the impact of COVID-19 on dengue specifically and provide forward-looking recommendations.

**Figure 3:**
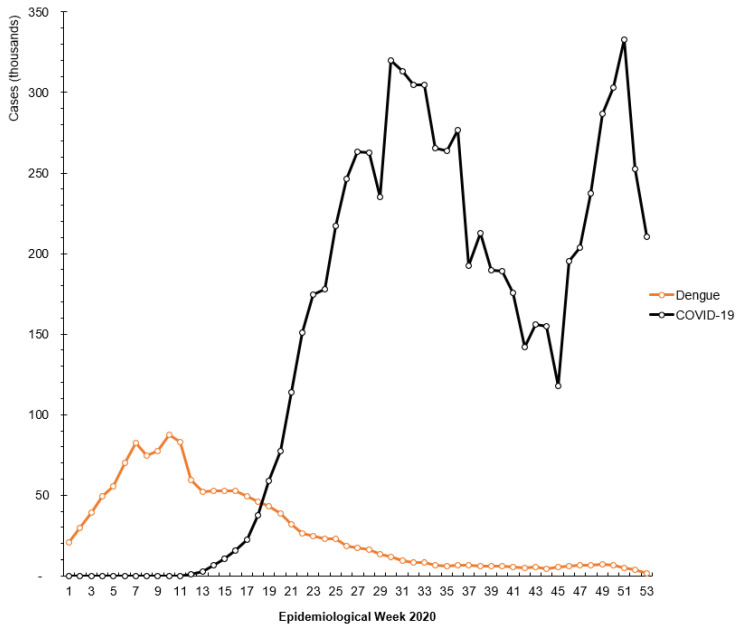
COVID-19 cases mapped against dengue cases (in thousands) by epidemiological weeks of the COVID-19 pandemic (from 1 January 2020).

One concern in early 2020 was the possibility of simultaneous dengue and COVID-19 outbreaks. When Brazil recorded its first case of COVID-19 in late February, the health system was already battling abnormally high levels of dengue cases in comparison to the previous year. However, this did not become a long-term reality as dengue cases declined after a peak in early March 2020. The epidemiological curves for both dengue and COVID-19 can be seen in Figure 3, highlighting how, while the outbreaks overlapped, their trajectories were quite different.

The steady but consistent decline in dengue cases several months into 2020 can be tied, in part, to the impact of COVID-19. As described above, the dynamics of dengue transmission and incidence are linked to seasonal rainfall, of which we see the most during the hotter and wetter months and less during the cooler and drier winter months. Complementing these seasonal changes was the ramping up of COVID-19 prevention measures and testing, which we believe may also have had an impact on dengue incidence. Specifically, the mobilization of existing epidemiological surveillance teams in response to the evolving COVID-19 situation caused a gap in resources dedicated to other diseases, resulting in a delay or underreporting of dengue cases. Additionally, the similarities in the clinical presentation of COVID-19, dengue, and other arboviruses could have been another contributing factor. Table 2 provides a comparison of common clinical manifestations for COVID-19, DENV, ZIKV, and CHIKV to highlight the difficulties in accurately identifying the condition, which can lead to misdiagnoses if there is no appropriate testing capacity to differentiate them.

Patients presenting with certain symptoms could have been tested for COVID-19, as the disease of priority, and negative test results were not followed up on to provide a proper diagnosis, leading to further underreporting. Underreporting and cross-reactivity of COVID-19 and arboviruses are closely linked. A study in the state of Espírito Santo using samples of patients who tested positive for dengue or chikungunya from 1 December 2019 to 30 June 2020 and who did not exhibit a previous history of COVID-19 retrospectively found that 2.85% of samples were actually positive for COVID-19. The study suggested “undiagnosed cases of COVID-19 may be prior to February 2020, and that these undiagnosed missed cases may have contributed to the fast expansion of SARS-CoV-2 outbreak in Brazil” [83]. Finally, based on what is known about health behaviors during the COVID-19 pandemic, we also believe individuals were less likely to go into the health clinics to get tested upon developing symptoms of dengue for fear of COVID-19 exposure. In this sense, COVID-19 may not have impacted the true incidence of dengue but rather the rate and quantity at which cases were reported [82]. This highlights a temporal element in how infectious disease reporting has changed through the pandemic, with underreporting COVID-19 masked by other arboviruses in the early months of the pandemic, followed by underreporting of arbovirus cases once COVID-19 was prioritized.

However, the literature suggests that the advent of social distancing, stay-at-home orders, and reduction in mobility across Brazil could have changed the epidemiological spread of dengue. Since the introduction of dengue into new communities is achieved through infected individuals traveling, as mosquitoes can only move short distances, a reduction in mobility—especially between rural and urban but also between urban and urban areas—could have had a true impact on dengue dispersion [84].

Looking forward, there is the possibility that other emerging infectious diseases will re-emerge with full force post-COVID. Following the Zika epidemic in Brazil, there was a decline in Zika cases in 2017 and 2018, mirrored by a decline in reported dengue cases during the same period, before resurging in unexpected proportions in 2019. The decline has been associated with protective immunity from ZIKV and DENV and environmental factors, while the resurgence has been attributed to new lineages of DENV, which had been circulating at low levels and erupted once conditions were prime [85]. While COVID-19 is a coronavirus, the changes in epidemiology that are expected could have an impact on future spread and lineage of dengue. On a resource level, the COVID-19 pandemic has hampered dengue prevention provisions on insecticide, which could have a very real impact on future vector distribution. According to the Brazilian Ministry of Health, vector control measures have been delayed due to COVID-19, including insecticide distribution, monitoring conducted by health agents, home visits, and community awareness programs around the dangers of stagnant water [86].

Another impact to note has been the “COVIDization” of research [87], which has seen funders and researchers pivot en masse to funding and studying COVID-19. While it is critical to support research in response to emerging situations, reactionary funding and research come at the cost of future pandemic preparedness [88]. There is a concern that dedicating too much attention to COVID-19, such as diverting funding and pivoting projects, could mean slow progress in necessary research for other emerging and neglected diseases such as dengue. Pandemic preparedness is key to averting future pandemics, meaning that continuous research into our known and unknown threats must be ensured, and must not be overcome by reactionary policies and oversaturation of research into the disease of the moment.

Finally, did Brazil learn from previous lessons? Did COVID-19 impact dengue? What can be learned from all of this? Brazil’s epidemiological surveillance and notification systems are proof that previous outbreaks have, in part, prepared the system for future public health emergencies. However, there were some pitfalls, namely: the lack of diagnostic capacity (despite this being a recommendation from PAHO to its members following the Zika outbreak), the lack of coherent policies and political discourse between state and federal governments, and the underfunded and overwhelmed SUS. Evidently, COVID-19 has impacted dengue on a number of different fronts, the extent of which we will not know for some time. Forward-looking recommendations include placing more emphasis on elevating standards for at-risk groups to mitigate circumstances that foster transmission and ensuring an adequately funded healthcare system, which can pivot resources to react to surges and waves of cases. More specifically, there is a need for robust country-wide testing capacity, including in low-resource settings. A testing regimen that could cover multiple infectious diseases would be essential, whether through a process of elimination using multiple tests or developing a diagnostic platform that can differentiate individual infections and families, e.g., coronaviruses and arboviruses. As for the future, pandemic preparedness must be the preferred approach to a pandemic response, which may come in the form of investing in the healthcare system, investing in the people to lift them out of poverty, and investing in research to anticipate the next threat.

## Figures and Tables

**Table 1:** Confirmed COVID-19 cases and deaths by state as of 4 November 2021, Brazil.

State	Region	Cases	Deaths	% of Cases	% of Deaths
São Paulo	Southeast	4,407,756	152,098	20	25
Minas Gerais		2,186,321	55,613	10	9.1
Rio de Janeiro		1,322,861	68,391	6.1	11
Espírito Santo		608,208	12,941	2.8	2.1
**Subtotal**	**Southeast**	**8,525,146**	**289,043**	**39**	**48**
Paraná	South	1,558,616	40,542	7.1	6.7
Rio Grande do Sul		1,468,595	35,525	6.7	5.8
Santa Catarina		1,218,044	19,697	5.6	3.2
**Subtotal**	**South**	**4,245,255**	**95,764**	**19**	**16**
Goiás	Central-West	906,441	24,211	4.2	4.0
Mato Grosso		544,094	13,694	2.5	2.3
Distrito Federal		515,399	10,902	2.4	1.8
Mato Grosso do Sul		376,558	9646	1.7	1.6
**Subtotal**	**Central-West**	**2,342,492**	**58,453**	**11**	**9.6**
Bahia	Northeast	1,246,278	27,091	5.7	4.5
Ceará		943,738	24,499	4.3	4.0
Pernambuco		632,366	20,033	2.9	3.3
Paraíba		454,529	9430	2.1	1.6
Rio Grande do Norte		373,926	7401	1.7	1.2
Maranhão		361,260	10,240	1.7	1.7
Piauí		326,089	7108	1.5	1.2
Sergipe		278,520	6031	1.3	1.0
Alagoas		240,360	6298	1.1	1.0
**Subtotal**	**Northeast**	**4,857,066**	**118,131**	**22**	**19**
Pará	North	598,703	16,748	2.7	2.8
Amazonas		427,867	13,772	2.0	2.3
Rondônia		270,767	6574	1.2	1.1
Tocantins		228,874	3882	1.0	0.6
Roraima		127,417	2030	0.6	0.3
Amapá		123,695	1993	0.6	0.3
Acre		88,053	1845	0.4	0.3
**Subtotal**	**North**	**1,865,376**	**46,844**	**8.5**	**7.7**
**Grand total for Brazil as at 4 November 2021**		**21,835,335**	**608,235**	**100**	**100**

**Table 2:** Common symptoms of mild cases of COVID-19, DENV, CHIKV, and ZIKV.

	COVID-19 ^^^	Dengue	Chikungunya	Zika
Average incubation period (days)	5–14	4–10	4–7	3–14
**Symptoms**				
Fever	✓	✓	✓	✓
Headache	✓	✓	✓	✓
Muscle and joint pain	✓	✓	✓	✓
Rash		✓	✓	✓
Tiredness	✓	✓	✓	✓
Nausea		✓	✓	
Cough	✓			
Loss of taste	✓			
Sore throat	✓			
Conjunctivitis				✓
Pain behind eyes		✓		

Note: This table is based on common symptoms and is not exhaustive. It characterizes symptoms of mild cases, which are more common and more ambiguous and therefore harder to accurately diagnose. ^ COVID-19 symptoms are based on common symptoms independent of variant. Source: WHO factsheets.

## Data Availability

This case study is a descriptive and analytical review of scientific literature supplemented with gray literature and news sources. No new data were collected for this study; all numbers and figures use publicly available data. Data sharing is not applicable to this article.
